# Isolated pulmonary valve endocarditis with rapid progression: a case report and literature review

**DOI:** 10.1186/s13019-020-01375-w

**Published:** 2021-01-28

**Authors:** Ming-Xuan Zhang, Wei-Min Zhang, Chan Yu, Bo-Wen Zhao, Ran Chen, Mei Pan, Bei Wang

**Affiliations:** 1 Department of Diagnostic Ultrasound and Echocardiography, Sir Run Run Shaw Hospital, Zhejiang University School of Medicine, No.3 East Qingchun Road, Hangzhou, 310016 China; 2 Department of Cardiac Surgery, Sir Run Run Shaw Hospital, Zhejiang University School of Medicine, Hangzhou, China

**Keywords:** Infective endocarditis, Pulmonary valve, Pulmonary embolism, Surgery, Case report

## Abstract

**Background:**

Isolated pulmonary valve endocarditis (IPE) is rare, accounting for 1.5–2% of all cases of infective endocarditis. Herein, we describe a case of isolated pulmonary valve endocarditis with rapid progression in a 28-year-old male. Unlike most patients reported previously who were cured with only anti-infective therapy, without surgery at an early stage, multiple complications occurred in this patient in less than 2 weeks.

**Case presentation:**

The patient was diagnosed with pulmonary valve endocarditis with blood cultures showing *Staphylococcus aureus* and echocardiography revealing 2 masses (measuring 14*13 mm、11*16 mm in size). Only 12 days later, acute massive pulmonary embolism occurred. Then, repeated echocardiography revealed multiple masses attached to the pulmonary valve with severe pulmonary insufficiency and the possibility of pulmonary valve destruction. Finally, pulmonary valve replacement, vegetation removal, and right pulmonary thromboendarterectomy together with resection of the middle and lower lobes of the right lung were performed.

**Conclusions:**

The role of surgery at an early stage might need to be reconsidered, and it may be viable to combine medical and surgical approaches.

## Background

Isolated pulmonary valve endocarditis (IPE) represents 1.5–2% of all cases of infective endocarditis [1] (IE). Intravenous drug abuse, sepsis, and central venous catheter or pacemaker implantation account for the majority of risk factors [2]. Most data concerning pulmonary valve (PV) infective endocarditis are mainly reported in small case series and case reports, some of which were published 25 years ago. Herein, we describe a case of IPE in a 28-year-old male complicated by rapid progression and multiple pulmonary emboli. Unlike most patients reported previously who were cured with only anti-infective therapy, multiple complications occurred in this patient in less than 2 weeks.

## Case presentation

A 28-year-old male was referred to the emergency department coupled with fever and chills for 2 weeks and was diagnosed with infective endocarditis in another hospital. He reported that he had traveled for 2 weeks across deserts in the United States and accidentally tore the skin of his left ankle 2 weeks ago. There was no history of diabetes or hypertension and no family history. Then, he presented to a hospital in the US and was treated with ibuprofen for high fever (value unknown); however, his body temperature normalized for a few hours and then rose again, with decreased appetite. With a body temperature of 40.3 °C, he was admitted to a hospital in Beijing 10 days prior. Investigations revealed a white blood cell (WBC) count of 14.30*10^9^/L, urine WBC count of 106.8/μl, urine red blood cell count 34.9/μl, and urine culture bacterium count of 203.2/μl, and repeated blood cultures showed *Staphylococcus aureus*. Chest CT (computed tomography) showed multiple inflammatory changes in both lungs, so the patient was started on anti-infective therapy with ceftriaxone and clarithromycin. During the treatment, fever occurred again, and his body temperature ranged from 38.6 to 40.3 °C, with cough and white phlegm. An echocardiogram revealed 2 masses (measuring 14*13 mm、11*16 mm in size), that were very mobile and appeared to be attached to the pulmonary valve. Then the patient was referred to our hospital with a WBC count of 16.4 × 10^9^/L, and a CRP(C-reactive protein) level of 113.2 mg/L, and echocardiogram (just 1 day later than the previous one) showed a mass attached to the pulmonary valve, which is measured 43.8*19.9 mm in size. Chest radiology revealed that multiple patchy and large lesions with cavities in both lungs. Therefore, the patient was diagnosed with endocarditis, septicemia and pulmonary infection, and was admitted to our hospital.

**Fig. 1:**
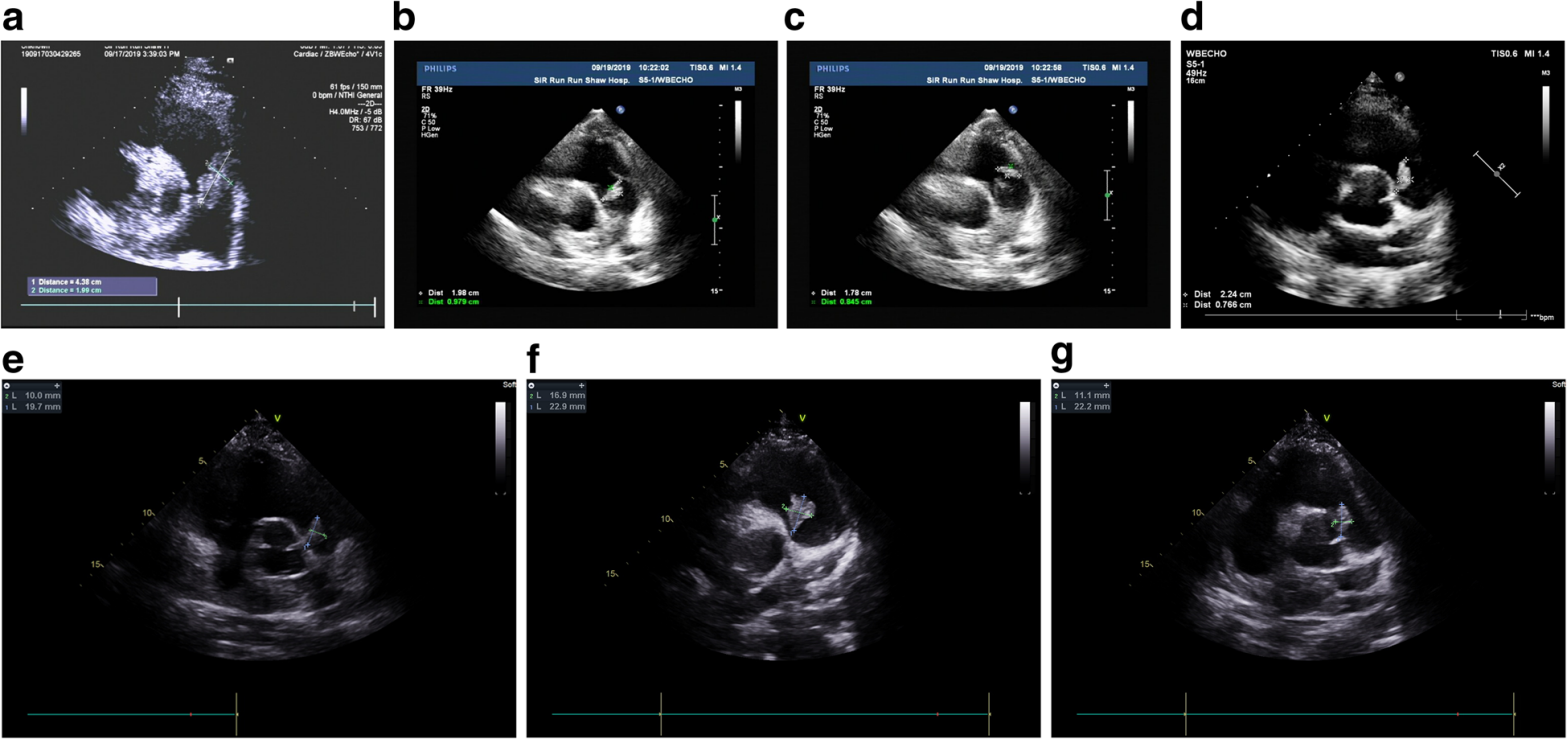
An ultrasonography series of ultrasonography showing PV vegetation from 1 day before admission to 2 days before the operation. Multiple cases of vegetations were observed, and one is measured in b-g. **a** was performed 1 day before admission, and **b** and **c** were performed on day 1 after admission. **d**, **e**, **f** and g were performed on days 8, 12, 16 and 26, respectively

**Fig. 2:**
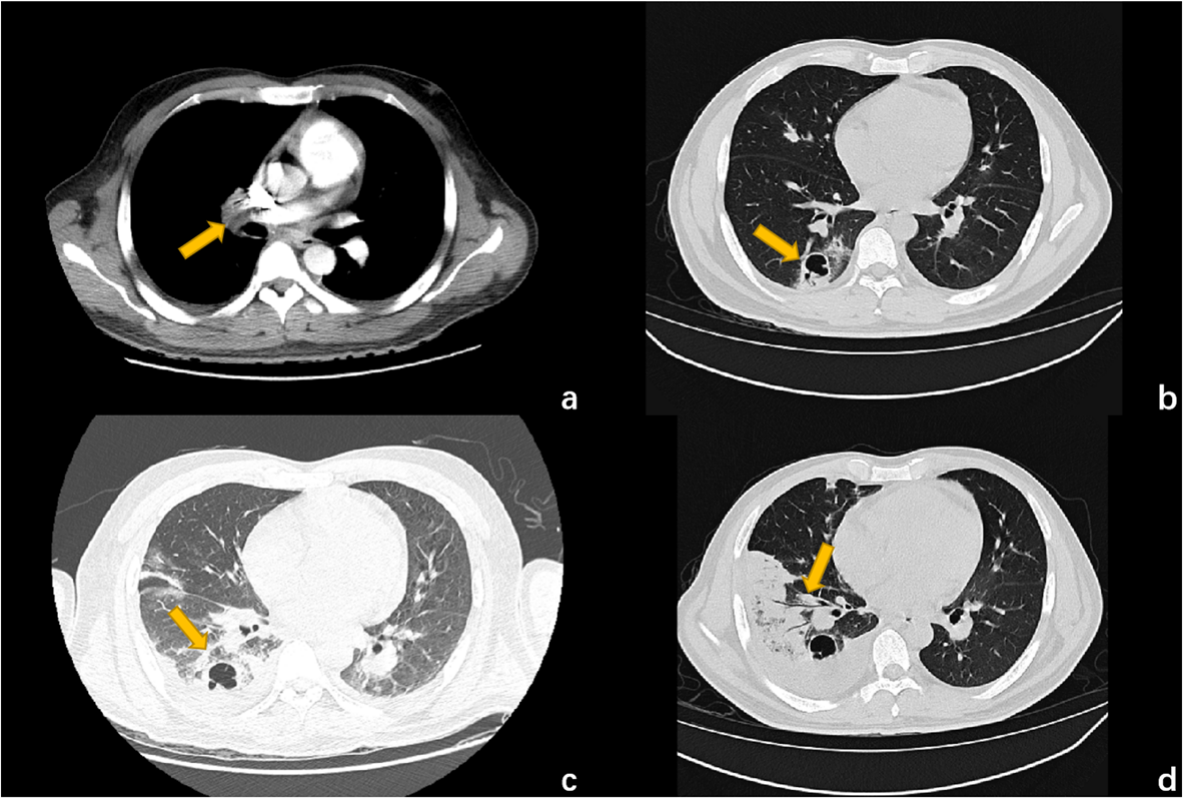
**a**. Computed tomographic pulmonary angiography on day 2 after admission. The arrow indicates the filling defect of the right pulmonary artery trunk. **b-d**. Chest computed tomography image on days 1, 5 and 27. Arrows indicate the cavity and inflammatory lesions in the right lung

**Fig. 3:**
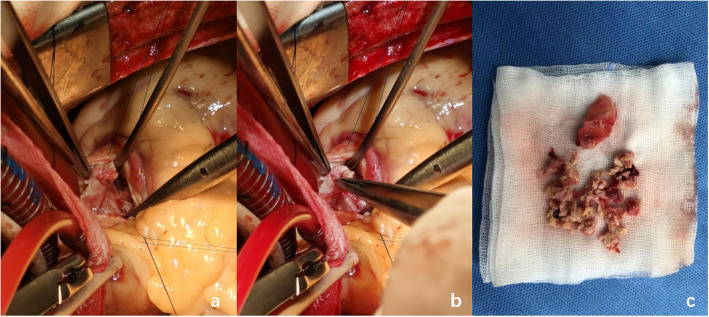
**a**, **b** Intraoperative photograph demonstrating the destroyed pulmonary valve leaflets and vegetation. **c** Destroyed pulmonary valve leaflets and vegetation

Given the patient’s age and short antibiotic treatment duration, valve replacement surgery was not the priority option and the patient wished to reconsider the surgery, so the treatment was mainly anti-infective and supportive therapy. Two days later, the patient reported chest tightness and dyspnea with increased DDi (D-dimer) (5.60 μg/mL) after using the toilet, and CTPA (computed tomographic pulmonary angiography) revealed filling defects of the right pulmonary artery and its branches as well as some branches of the left pulmonary artery, and a pulmonary embolism was considered. Therefore, the patient was transferred to the ICU (intensive care unit), and interventional pulmonary artery thrombectomy was immediately conducted. After 1 week of treatment in the ICU, the patient was transferred to the cardiac surgery department to prepare for valve replacement surgery, as the last echocardiogram showed multiple masses attached to the pulmonary valve with severe pulmonary insufficiency and the possibility of pulmonary cusp destruction (Fig. 1). Nevertheless, repeated blood culture was negative. With the strike of both infection and pulmonary embolism, the patient’s hemachrome and albumin levels continued to decrease. Moreover, together with the recurrent fever and an increase in inflammatory indicators, the patient was too vulnerable to undergo surgery, so anti-infective therapy was conducted temporarily utill the patient was in a stable condition. The latest CTPA and chest CT results showed a right lower pulmonary embolism and right lower lobe pneumonia (Fig. 2). Twenty days later, given the poor effect of the anti-infective treatment and the strong operation desire of the patient, pulmonary valve replacement, vegetation removal and right pulmonary thromboendarterectomy were performed. Destroyed pulmonary valve leaflets with friable vegetation measuring 2.5*1.0 cm in size and gray vegetation in the right pulmonary artery were revealed (Fig. 3). Radical debridement with valve excision was performed, and then the St. Jude bioprosthetic valve (size 23 mm) was closed with interrupted sutures. Under direct vision through an incision into the right pulmonary artery, the thrombus was removed with a double-lumen catheter. During the operation, bronchoscopy showed that blood filled all levels of the bronchus on both sides, and active hemorrhage occurred in the distal right middle bronchus. After a brief multidisciplinary discussion, resection of the middle and lower lobes of the right lung was conducted.

Histopathology revealed infectious destruction in the specimen from the pulmonary valve and extensive hemorrhage and infarction of lung tissue with inflammatory cell infiltration. The patient’s postoperative course was uneventful, and his laboratory parameters were all normalized before his discharge. He was discharged on the 30th postoperative day. The patient continued antibiotic therapy for 1 month after the operation. The mean peak systolic transvalvular gradients were 19 mmHg, 24 mmHg and 23 mmHg on postoperative days 8, 23 and 85, respectively, according to echocardiography.

## Discussion and conclusion

Right-sided IE, especially IPE is rare, occurring ten times less frequently than tricuspid valve endocarditis [1]. A structurally normal pulmonary valve is hardly affected alone. The first possible reason is that the lower pressure gradient through the pulmonary valve results in less shear stress than other valves. This leads to less valvular damage and protects the PV from IE occurrence. Second, valvular abnormalities are rare in the PV.

In a prospective cohort study, the main pathogenic microorganism isolated from blood culture was gram’s bacteria (83%), of which *Staphylococcus aureus* accounted for 31% [3]. The most common pathogenic microorganism in North America is *Staphylococcus aureus* [3, 4], which is consistent with the patient’s history and blood culture.

In the review of our patient, a rapidly progressive course was observed. The patient was diagnosed with bacteremia 4 days after his first fever and went on antibiotic therapy. The blood culture results were obtained on the 7th day, and antibiotics were adjusted according to these results. On the 12th day, vegetation was discovered and measured 14*13 mm and 11*16 mm in size, while 1 day later, the whole vegetation measurement was 43.8*19.9 mm. Then, the patient was transferred to the ICU for pulmonary embolism on the 16th day. Most embolism events occur within 2–4 weeks of antibiotic therapy [5]. In this case, pulmonary embolism occurred in less than 2 weeks. IE due to Staphylococcus spp. was found to be an independent predictor of worse in-hospital outcomes [6], and Staphylococcus spp. was an independent predictor of in-hospital mortality, which has been confirmed to be associated with worse prognosis [3, 7, 8]. This may be one of the reasons for the rapid decline in our patient’s statues. Furthermore, except for the first blood culture, the repeated cultures remained negative as the situation deteriorated rapidly, which made the treatment more complicated.

As the AHA (American Heart Association) guidelines [9] recommend, both TTE (transthoracic echocardiography) and TEE (transesophageal echocardiography) are indispensable in many patients with IE during initial evaluation and subsequent follow-up, and they provide complementary information. It is estimated that the sensitivity and specificity of TTE are 30–63% and 91–100%, respectively, and those of TEE are 87–100% and 91–100%, respectively [10] Even if our patient’s blood culture remained negative, TTE would provided additional information to evaluate the severity of IE. Robbins et al. found that vegetation size could predict the response to medication alone [11] The response to medication of vegetations < 10 mm was 100% versus 63% in vegetaion > 10 mm, and surgery was unavoidable for the remaining patients. In their assumption, as bacterial colonies deepen, their metabolism and proliferation become slower, leading to certain antibiotics being less effective. All this evidence indicates that our patient might benefit more from surgery than from conservative treatment alone.

However, it seems that surgery is not the optimal treatment for right-sided IE. The AHA guidelines [9] recommend that right-sided IE should be treated as conservatively as possible, and nonrandomized trial data from a single center experience [12] and international collaboration [13] support that early valve surgery may not be beneficial for all primary patients with primary IE caused by *Staphylococcus aureus*. Even the ESC (European Society of Cardiology) guidelines do not explain the role of surgery in pulmonary valve infection [14]. This unclear attitude may be due to the rarity of occurrence and invasiveness of right-sided IE [15] However, embolization, valve destruction, and large vegetation were indicated in our patient, and the general situation deteriorated in a short time. The role of surgery at an early stage in patients with such a rapid course and multiple complications, might need to be reconsidered. As Witten JC et al. found in a 13-year retrospective study of right-sided IE [4] if surgery was performed at an early stage, the surgical risk was low. Otherwise, with the burden of septic pulmonary embolism, the risk increases, and the opportunity for intervention may narrow as pulmonary complications result in a rapid decline in patient status, which is similar to our patient. Recurrent IE was is infrequent in their study (the greatest occurrence was found in injection drug users). Even if pulmanory IE only accounts for 5% of cases, it provides clues to support IPE surgery in the early course. Given the 15-year single-center experience from Liekiene D et al. [16], removal of vegetaion by preserving the valve is the most beneficial at the early stage of IPE [16, 17]. However, as surgery is not commonly recommended at an early stage, pulmonary cusps are damaged when surgeons see the patients. In this case, the most common method is pulmonary valve replacement. Moreover, the most significant point from their study is that surgery performed earlier may make the surgery less radical, and early surgery may improve patient outcomes, which is worth learning in cases such as our patient.

Postoperative results are generally favorable, as two of the largest case series reported that none of the nine cases had repeated vegetation [17, 18] The bioprosthetic valve of our patient functioned well and stably after 3 months of follow-up. As with some reports supporting early surgical interventions, it may be viable to combine medical and surgical approaches in IE patients upon admission.

## Data Availability

Authors do not wish to share the data because that it might identify the patient.

## Abbreviations

IPE: Isolated pulmonary valve endocarditis
IE: Infective endocarditis
PV: Pulmonary valve
WBC: White blood cell
CT: Computed tomography
CRP: C-reactive protein
DDi: D-Dimer
CTPA: Computed tomographic pulmonary angiography
ICU: Intensive care unit
AHA: American Heart Association
ESC: European Society of Cardiology
TTE: Transthoracic echocardiography
TEE: Transesophageal echocardiography
